# Serum Amino Acid Profile Changes After Repetitive Breath-Hold Dives: A Preliminary Study

**DOI:** 10.1186/s40798-022-00474-3

**Published:** 2022-06-20

**Authors:** Danilo Cialoni, Andrea Brizzolari, Nicola Sponsiello, Valentina Lancellotti, Gerardo Bosco, Alessandro Marroni, Alessandra Barassi

**Affiliations:** 1 Environmental Physiology and Medicine Laboratory, Department of Biomedical Sciences, University of Padua, Padua, Italy; 2 DAN Europe Research Division, Contrada Padune 11, 64026 Roseto degli Abruzzi, TE Italy; 3 Apnea Academy Research, Padua, Italy; 4 Cardiothoracic and Vascular Department, Azienda Ospedaliero-Universitaria Pisana (AOUP), Pisa, Italy; 5 Department of Health Science, University of Milan, Milan, Italy

**Keywords:** Breath-hold, Diving, Amino acid, Hypoxia, Physical activity

## Abstract

**Background:**

The aim of this work was to investigate the serum amino acid (AA) changes after a breath-hold diving (BH-diving) training session under several aspects including energy need, fatigue tolerance, nitric oxide (NO) production, antioxidant synthesis and hypoxia adaptation. Twelve trained BH-divers were investigated during an open sea training session and sampled for blood 30 min before the training session, 30 min and 4 h after the training session. Serum samples were assayed for AA changes related to energy request (alanine, histidine, isoleucine, leucine, lysine, methionine, proline threonine, valine), fatigue tolerance (ornithine, phenylalanine, tyrosine), nitric oxide production (citrulline), antioxidant synthesis (cystine, glutamate, glycine) and hypoxia adaptation (serine, taurine).

**Main results:**

Concerning the AA used as an energy support during physical effort, we found statistically significant decreases for all the investigated AA at T1 and a gradual return to the basal value at T2 even if alanine, proline and theonine still showed a slight significant reduction at this time. Also, the changes related to the AA involved in tolerance to physical effort showed a statistically significant decrease only at T1 respect to pre-diving value and a returned to normal value at T2. Citrulline, involved in NO production, showed a clear significant reduction both at T1 and T2. Concerning AA involved in endogenous antioxidant synthesis, the behaviour of the three AA investigated is different: we found a statistically significant increase in cystine both at T1 and T2, while glycine showed a statistically significant reduction (T1 and T2). Glutamate did not show any statistical difference. Finally, we found a statistically significant decrease in the AA investigated in other hypoxia conditions serine and taurine (T1 and T2).

**Conclusions:**

Our data seem to indicate that the energetic metabolic request is in large part supported by AA used as substrate for fuel metabolism and that also fatigue tolerance, NO production and antioxidant synthesis are supported by AA. Finally, there are interesting data related to the hypoxia stimulus that indirectly may confirm that the muscle apparatus works under strong exposure conditions notwithstanding the very short/low intensity of exercise, due to the intermittent hypoxia caused by repetitive diving.

## Key Points

- A breath-hold diving training session can change the serum amino acids under several aspects including energy need, fatigue tolerance, NO production, antioxidant synthesis and hypoxia adaptation.
- With the exception of cystine, all the investigated AA decreased after the end of the breath-hold diving session and most of them returned to the basal value some hours later.
- During BH-diving, AA may support some biological processes necessary to adapt the human body to the hyperbaric environment, including hypoxia. These data might confirm the hypoxia role in the muscle work despite the very short/low intensity of exercise, due to the intermittent hypoxia caused by repetitive dives.

## Introduction

Breath-hold diving (BH-diving), the first underwater activity practised by mankind [1], involves a complex adaptation mechanism, related to the increases in ambient pressure, called “diving response” that includes bradycardia, reduced cardiac output, increased arterial blood pressure, peripheral vasoconstriction and blood gases composition [2, 3]. The activation of the sympathetic and parasympathetic nervous system reduces the O_2_ consumption in peripheral tissues to ensure adequate O_2_ supply to vital organs [4] such as brain, heart, liver and skeletal muscles [5–8]. In BH-diving, while hyperbaric hyperoxia occurs at depth due to the increased environmental pressure and thorax compression [9], hypoxia appears in the ascending phase of a dive [10].

On the other hand, BH-diving is associated with a physical activity in particular conditions of intermittent hypoxia, this aspect can overcome the diving reflex [11] and can result in an increased exercise-induced skeletal muscle fatigue/stress that increases muscles injury markers such as creatine kinase (CK), cardiac creatine kinase isoenzyme (CK-MBm), brain natriuretic peptide (BNP), lactate dehydrogenase (LDH) [12, 13], copeptin and cortisol [12]. Furthermore, endothelial dysfunction, well investigated in BH-diving [14, 15], may lead to smooth muscle alteration.

The release of Amino Acids (AA) and their skeletal muscle catabolites in the blood circulation, and their reuptake by other tissues are parts of complex metabolic pathways aimed at maintaining energetic homeostasis [16]. As it is well known, AA are involved in several metabolic activities and particularly:

- *Fuel metabolism*: To ensure enough energy during exercise through the involvement of Alanine (ALA) and the three branched-chain amino acids (BCAA): Leucine (LEU), Isoleucine (ILE) and Valine (VAL). ALA is synthesized in skeletal muscle and released in the bloodstream [17] to be transported to the liver where to be regenerated into pyruvate. BCAA are the most relevant AA metabolized during physical exercise for energetic request (because physical activity needs energy substrates) [18]. BCAA may protect from protein degradation and muscle enzyme release [19] reducing skeletal muscle damage during prolonged physical effort [20], mitigating central fatigue [21] and promoting recovery of muscle function [22]. During physical activity also Hystidine (HIS), Threonine (THR), Lysine (LYS) and Methionine (MET) are involved in catabolic processes (Krebs Cycle) [23] to produce the necessary energy to sustain the effort. Proline (PRO) can be converted in α-ketoglutarate, a Krebs cycle intermediate to sustain the energy request. Furthermore, PRO may be involved in to the free fatty acids (FFA) catabolism [24, 25].
- *Improved exercise fatigue tolerance*: Physical activity increases catecholamine levels in athletes, depending on duration and intensity of exercise [26]. Some aromatic amino acids, Tyrosine (TYR), Phenylalanine (PHE) can influence the levels of catecholamine precursors [27]: plasma catecholamine precursors are associated with improved tolerance during prolonged physical exercise [28]. Ornithine (ORN) promotes lipid metabolism, activates the urea cycle, and improves fatigue tolerance ameliorating physical performance [29].
- *Nitric oxide (NO) production*: NO is produced by the conversion of Arginine into Citrulline (CIT) by nitric oxide synthase enzymes (NOS) and it reflects NO synthesis [30]. During physical exercise also the catecholamines stimulate the production of NO [31] that plays a key role in skeletal muscle contractile function [32], increasing in response to acute sessions of exercise [33–35] to adapt the body to exercise training [36]. NO plays a key role in the response to hyperbaric exposure, modulating the endothelial adaptation during BH-diving [37, 38].
- *Antioxidant defences*: A prolonged strenuous physical activity can lead to oxidative stress with the generation of reactive oxygen species (ROS) and reactive nitrogen species (RNS) [39] reducing NO availability [40]. Human body can activate antioxidant defences to protect tissue from the toxic effects of free radicals [41]. Cystine (CYST), Glutamate (GLU) and Glycine (GLY) are necessary for the glutathione biosynthesis [42], a component of the body antioxidant defences. Endogenous antioxidants protect macromolecules from vascular oxidative stress due to increased O_2_ level associated with hyperbaric condition [43] and hyperoxic exposure, as observed by data obtained from an underwater blood draw (− 42 m) carried out on SCUBA [44] and BH-divers [37].
- *Hypoxic response*: There are little knowledge on the release of AA in case of hypoxic exposures due to environmental variations or diseases (e.g. cancer and chemical hypoxia); Serine (SER) [45] and Taurine (TAU) [46] seem involved in hypoxia conditions. Changes in AA in BH-diving were studied in high altitude showing an increase in ALA and BCAA, representing an potential adaptation mechanism to hypoxia [47].

The aim of the present study is to investigate the serum AA profile changes after repetitive BH-dives in elite athletes.

## Material and Methods

### Subjects and Diving Protocol

A total of 12 expert healthy BH-divers were investigated during an open sea training session at Elba Island, Italy. All the divers were informed about risks and benefits of this study, read and signed a specific informed consent form before the experiment and provided personal anthropometric parameters. The study was conducted in accordance with the Helsinki Declaration, authorized by Ethical Committee of Università degli Studi di Milano (Aut. n° 37/17).

The selected volunteers are labelled “expert” because they are affiliated to the “Apnea Academy” Training Agency as instructors or high-level BH-divers and are able to reach-a minimum of 30 m in constant weight, 4 min static apnoea (at the surface), 75 m dynamic BH-diving (horizontal) in a swimming pool (distance).

The exclusion criteria were history or clinical evidence of hypertension, cardiac, pulmonary, or any other significant disease; any acute illness during the 15 days before the experiment; use of aspirin, paracetamol, or other anti-inflammatory drugs in the 7 days before the experiment; compressed-gas diving during the 30 days before the test.

As per Apnea Academy standard procedures, all the divers were asked to perform a number of dives at increasing depth with a surface interval at least 3/4 the diving time to do a gradual approach to the maximum daily personal depth. When ready, they performed the last dive reaching the maximum depth of the training session. All the dives were performed as constant weight bi-fins discipline (CWTB). Divers dressed in a 5 mm wetsuit.

Diving profiles, including mean depth, maximum depth, and number of dives, were recorded using a free-diving computer (UP-X1 Omersub Spa, Monza Brianza, Italy). This computer measured and recorded diving data every 2 s.

The diving profile showed a mean number of dives of 17.7 ± 3.2; a mean depth of 32.5 m ± 6.1 m; a mean of maximum depth of 42.9 m ± 1.7 m.

### Blood Draw Protocol

A butterfly needle (21G × 34 0.8 × 19 mm Green) was placed in the antecubital vein to collect 5 ml of blood using 5 ml serum containing tube (Vacutainer, Becton, Dickinson and Company, Franklin Lakes, NJ, USA). We collected blood per each of the following time steps:

- Basal: 30 min before the start of the warm-up;
- T1: 30 min after the end of dive sessions;
- T2: 4 h after the end of dive session.

After 15 min and before 30 min from collection at room temperature, blood samples were centrifuged (3000 rpm for 10 min) to separate serum from cell fraction and were frozen at − 20 °C. Then, the serum samples were then delivered to the laboratory and kept at − 20 °C until the analysis.

### Serum Amino Acid Analysis

The concentrations of the following AA were measured in serum:

- ALA, HIS, ILE, LEU, LYS, MET, PRO, THR, VAL, as concerning the energy need;
- TYR, PHE, ORN as concerning the tolerance to the prolonged physical activity;
- CIT as concerning NO production;
- CYST, GLU, GLY as antioxidant synthesis;
- SER, TAU as concerning the BH-diving related hypoxia.

Serum AA concentrations were determined by a Biochrom30plus Amino Analyzer (Biochrom Ltd., Cambridge, UK, EU), a cation-exchange chromatography system [48]. Briefly, AA were purified mixing the serum samples 1:1 v/v with 10% sulfosalicylic acid (Sigma-Aldrich Corp., St. Louis, MO, USA) containing the internal standard norleucine 500 μmol/L (Sigma-Aldrich Corp.) and adding 2 volumes of lithium citrate loading buffer pH 2.20 [48]. After strong agitation and cooling at 4 °C for 5 min, the samples were centrifuged 8 min at 14,000 rpm. Supernatants were filtered and 100 μL were the operative injection volumes. Post-column derivatization with ninhydrin allowed the detection of AA at the wavelength of 570 nm, while 440 nm for Pro. Standard lithium citrate buffers with pH 2.80, 3.00, 3.15, 3.50, and 3.55 and ninhydrin reagents utilized during separation were provided ready to use by Biochrom Ltd. Briefly, 125 μmol/L AA standard solution was prepared by mixing physiological basis with acids and neutrals and internal standard solution (Sigma-Aldrich Corp.). Data analysis was performed by EZChrome software (Agilent Technologies, Santa Clara, CA, USA). Areas of the peaks were used to determine AA concentrations, and they were expressed in terms of μmol/L. All divers were let loose on their usual diet without any AA supplementation or conditioning in food, we measured the serum AA value before and after the training session studying the relative delta regardless of differences in diet.

Urine density, haemoglobin and haematocrit were also recorded and used to calculate changes in blood volume (BV), red cell volume (CV), and plasma volume (PV) before and after the dive series, using the Dill and Costill formula [49].

### Statistical Analysis

Data are presented as mean ± standard deviation (SD) for parametric data and median, or range for nonparametric data. The D’Agostino and Pearson normality test was used to assume a Gaussian distribution. Then, data were analysed by either the one-way ANOVA for multiple comparison, or the Friedman test for multiple comparison of parametric and nonparametric data, respectively. A probability lower than 5% was assumed as the threshold to reject the null hypothesis (*p* < 0.05). To minimize the subject-to-subject variability, data are normalized against the basal value (TO). We also investigate for correlation between diving profile (Maximum dept, mean of dept, N° of dives) and the reduction in AA involved in energy support.

Data were also analysed to find correlation between AA changes and dive parameters. But even in this case, a probability lower than 5% was assumed as the threshold to reject the null hypothesis (*p* < 0.05). A correlation is strong if *r* is ≥ 0.7 (positive correlation) or ≤  − 0.7 (negative correlation).

Data were analysed using Prisma GraphPaD 9 software.

## Results

A total of 12 experienced BH-divers, nine male and three female, with mean age 41.6 ± 5.6 years, mean height 178.6 ± 9.8 cm; mean weight 76.5 ± 12.8 kg and BMI 23.8 ± 2.2 were investigated.

All the volunteers completed the experiment without Taravana episodes, evidence of pulmonary and/or ear barotraumas or other health problems. Diving session was performed at Elba Island, Italy, in salt water at 21 ± 0.5 °C mean temperature, as recorded by diving computer.

Almost all the investigated AA showed a significant reduction after diving, some of them returned at pre-diving value at T2 (4 h). Only CYS, involved in endogenous antioxidant synthesis, showed a significant increase after diving (T1 and T2).

**Fig. 1:**
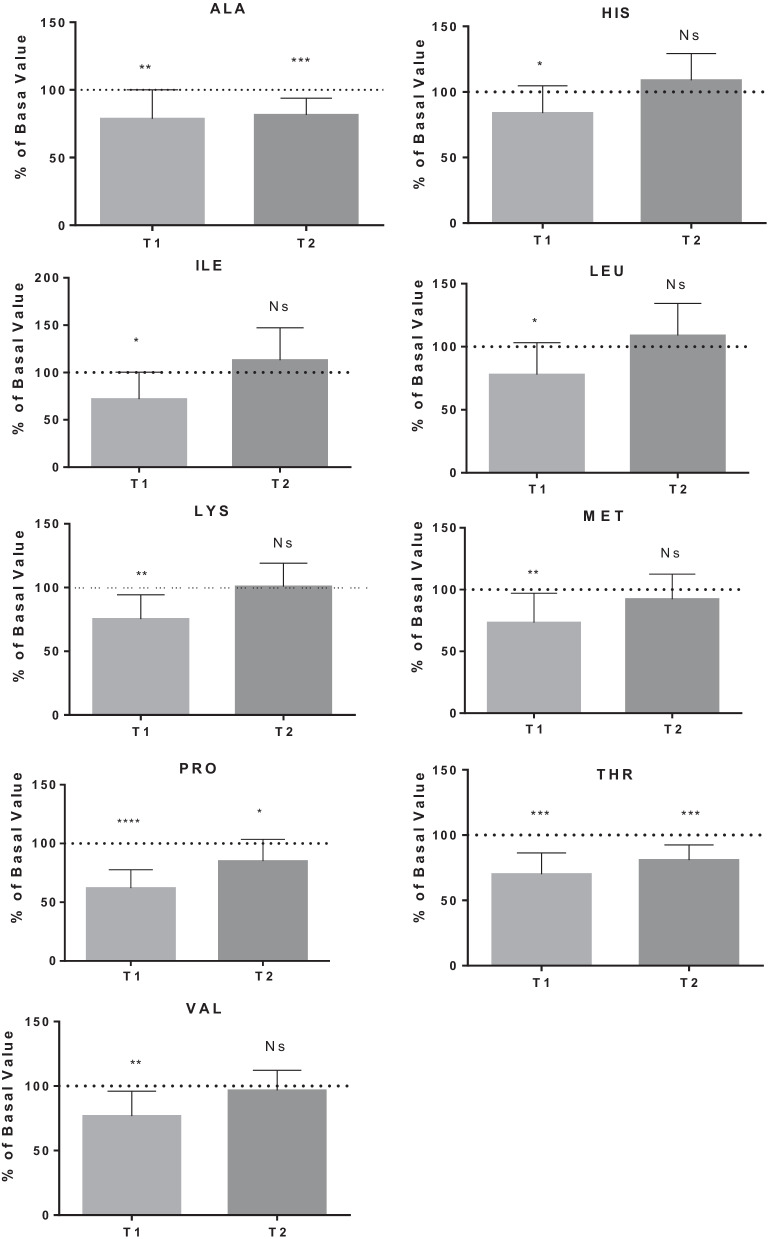
AA changes related to energy need. Data are expressed as percentage of control value. Asterisks indicate a value significantly different compared to basal (*p < 0.05, **p < 0.01, ***p < 0.001, ****p < 0.0001)

**Fig. 2:**
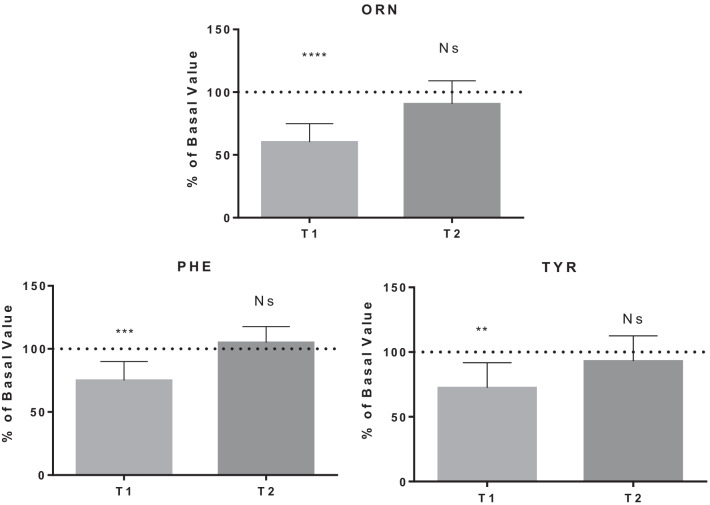
AA changes related to tolerance of physical effort

**Fig. 3:**
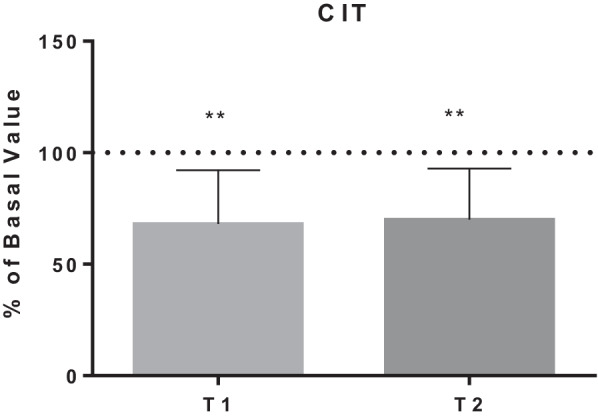
AA changes related to NO production

**Fig. 4:**
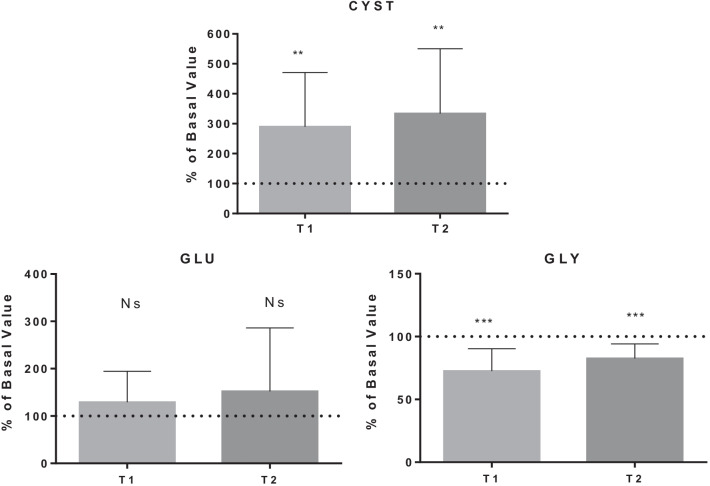
AA changes related to antioxidant synthesis

**Fig. 5:**
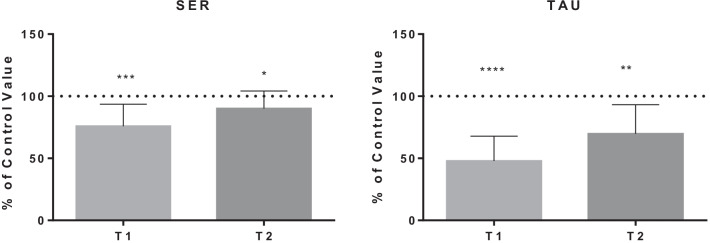
AA changes related to hypoxia tolerance

The detailed results are shown in Figs. 1, 2, 3, 4 and 5, while Table 1 shows the details for each investigated AA.

**Table 1:** AA changes in BH-divers

Amino acid	Basal value (µmol/l)	T1 value (µmol/l)	T1% of basal value	T2 value (µmol/l)	T2% of basal value	*P* value
*Energy need*						
Alanine	454.0 ± 97.1	343.1 ± 69.1	78.7 ± 21.4	364.1 ± 64.2	81.5 ± 12.4	T1:**/T2:***
Histidine	70.5 ± 11.6	57.6 ± 11.2	83.9 ± 20.8	75.4 ± 10.1	108.9 ± 20.4	T1:*/T2: Ns
Isoleucine	70.7 ± 24.8	46.2 ± 14.5	71.9 ± 28.6	75.6 ± 21.2	113.0 ± 34.3	T1:*/T2: Ns
Leucine	149.5 ± 40.8	110.1 ± 28.5	77.9 ± 25.2	157.3 ± 34.5	108.9 ± 25.4	T1:*/T2: Ns
Lysine	175.8 ± 26.5	129.8 ± 28.7	75.3 ± 19.1	175.6 ± 32.0	100.8 ± 18.3	T1:**/T2: Ns
Methionine	28.3 ± 6.7	19.7 ± 4.7	73.4 ± 23.8	25.9 ± 7.6	92.3 ± 20.2	T1:**/T2: Ns
Threonine	167.1 ± 39.9	114.5 ± 27.8	70.1 ± 16.3	134.1 ± 31.9	81.0 ± 11.4	T1:***/T2:***
Proline	234.1 ± 76.2	143.8 ± 57.2	62.0 ± 15.6	198.8 ± 74.6	85.0 ± 18.5	T1:****/T2:*
Valine	257.1 ± 43.9	194.1 ± 48.0	76.8 ± 19.2	246.5 ± 47.8	96.6 ± 15.6	T1:**/T2: Ns
*Fatigue tolerance*						
Ornithine	85.6 ± 25.7	49.7 ± 13.8	60.4 ± 14.5	76.4 ± 24.8	90.7 ± 18.4	T1:****/T2: Ns
Phenylalanine	73.1 ± 14.3	53.3 ± 7.4	75.1 ± 14.9	76.7 ± 18.1	105.0 ± 12.7	T1:***/T2: Ns
Tyrosine	77.6 ± 18.1	54.9 ± 13.4	76.4 ± 19.4	70.2 ± 12.3	93.0 ± 19.6	T1:***/T2: Ns
*Nitric oxide production*						
Citrulline	25.8 ± 16.7	21.1 ± 5.4	68.1 ± 24.2	19.3 ± 9.6	70.0 ± 22.9	T1:**/T2:**
*Antioxidant synthesis*						
Cystine	8.7 ± 5.8	18.6 ± 7.1	289.6 ± 181.3	21.2 ± 6.8	333.9 ± 216.7	T1:**/T2:**
Glutamate	19.0 ± 11.4	19.6 ± 8.0	129.2 ± 65.3	22.2 ± 14.5	152.1 ± 134.2	T1: Ns/T2: Ns
Glycine	227.7 ± 59.0	160.4 ± 36.8	72.6 ± 17.7	186.1 ± 46.4	82.6 ± 11.6	T1:***/T2:***
*Hypoxia tolerance*						
Serine	138.4 ± 27.2	101.7 ± 16.6	75.8 ± 17.8	123.5 ± 26.5	90.1 ± 14.0	T1:***/T2:*
Taurine	121.2 ± 28.1	54.2 ± 17.2	47.8 ± 20.1	80.0 ± 16.0	69.6 ± 23.6	T1:****/T2:**

Data are expressed as percentage of control value. Asterisks indicate a value statistically significantly different compared to basal value (*p < 0.05, **p < 0.01, ***p < 0.001, ****p < 0.0001)

We did not find any differences in blood volume between pre- and post-diving value (*p* > 0.05).

We found a significant correlation between several AA involved in energy supply and mean of depth, especially at T1 with THR (*p* = 0.01; *r*: 0.80) and VAL (*p* = 0.01; *r*: 0.72). Table 2 shows the complete results.

**Table 2:** Correlation between AA involved in energy need and dive parameters at T1 and T2

Amino acid	T1						T2					
	Mean of depth		No of dives		Maximum depth		Mean of depth		No of dives		Maximum depth	
	*p*	*r*	*p*	*r*	*p*	*r*	*p*	*r*	*p*	*r*	*p*	*r*
Alanine	Ns	0.44	Ns	0.14	Ns	− 0.41	Ns	0.12	Ns	− 0.15	Ns	− 0.01
Histidine	0.02*	0.67	Ns	− 0.12	Ns	− 0.40	Ns	0.4	Ns	− 0.37	Ns	− 0.04
Isoleucine	0.04*	0.59	Ns	− 0.40	Ns	− 0.36	Ns	0.50	Ns	− 0.51	Ns	− 0.45
Leucine	0.02*	0.68	Ns	− 0.37	Ns	− 0.48	Ns	0.5	Ns	− 0.53	Ns	− 0.54
Lysine	0.02*	0.68	Ns	− 0.13	Ns	− 0.55	Ns	0.41	Ns	− 0.53	Ns	− 0.12
Methionine	Ns	0.55	Ns	− 0.15	Ns	− 0.25	Ns	0.25	Ns	− 0.57	Ns	0.12
Proline	0.04*	0.61	Ns	0.10	Ns	− 0.41	Ns	0.22	Ns	− 0.07	Ns	− 0.05
Threonine	0.01**	0.80	Ns	− 0.02	0.03*	− 0.62	Ns	0.45	Ns	− 0.40	Ns	− 0.27
Valine	0.01**	0.72	Ns	− 0.25	Ns	− 0.48	0.05*	0.58	Ns	− 0.42	Ns	− 0.44

*Ns* not statistically significant

^*^p < 0.05; **p < 0.01; ***p < 0.001; ****p < 0.0001

## Discussion

This study aimed at investigating serum AA changes related to BH-diving in elite BH-divers after an open sea training session under several aspects: energy need, fatigue tolerance, NO and antioxidant production, hypoxia tolerance.

The diving protocol was designed to expose all BH-divers to their maximum personal effort level during a “usual” free-diving training session, for which we did not impose any number of warm-up dives or maximum depth.

BH-diving-related physical effort can request an increase in catabolic metabolism to produce adequate amount of ATP. We observed a statistically significant decrease in several AA that can be used as substrate for energy need including ALA, LEU, ILE, VAL, HIS, THR, LYS, MET. ALA reduction can be the consequence of pyruvate production that is converted in acetyl-CoA to produce ATP (Krebs Cycle). ALA release should also lead to the muscle protein synthesis but, in this case, the Cahill cycle did not occur for the inhibition of proteosynthetic cascade [50].

During physical activity, BCAA (LEU, ILE,VAL) release, splanchnic bed rises and is accompanied by an elevated BCAA uptake by contracting muscles and by an enhancement of BCAA oxidation therein [18]. In skeletal muscle, BCAA oxidation is catalysed by branched-chain α-keto acid dehydrogenase (BCKDH) [51] to use them as energetic substrate [52] providing about 3–6% of the total energy demand [53], according to some authors that observed a decrease in BCAA after prolonged effort such as a tennis tournament [54], a marathon [55] and a cyclist race [56], and LEU decrease in sprinters and jumpers when the muscles work in anaerobic conditions [57].

HIS decrease can be explained because it is converted into GLU, then in α-ketoglutarate to go into the Krebs cycle. THR is converted to pyruvate via threonine dehydrogenase. An intermediate in the THR catabolism can undergo thiolysis with coenzyme A (CoA) to produce acetil-CoA.

LYS is the precursor for carnitine [58] which transports fatty acids to the mitochondria, where they can be oxidised to produce acetil-CoA, involved in tricarboxylic acid (TCA) cycle [59]. Finally, for this group, according to data obtained by other authors [60], MET decreased after prolonged physical activity. This reduction may reflect increased transmethylation in which DNA, histones and other macromolecules are methylated in response to exercise [60]. PRO may also be related to the free fatty acids (FFA) release because some authors found a correlation between the decrease in PRO and the increase in FFA [25] as energy source during prolonged physical activity.

Physical activity led to release of antifatigue molecule precursors to improve the tolerance to physical effort [28]. TYR, obtained from PHE, is decomposed to give acetoacetate and fumarate that go into the TCA cycle. Its slow recovery is due to the PHE reduction: PHE is used to produce catecholamines, as observed by Sponsiello et al.: urine catecholamine levels increased immediately after the dives, while we would have expected despite the participants were very expert BH-divers [61].

TYR decrease after the BH-diving session: this may be related to the stimulation of catecholamines (dopamine, norepinephrine, epinephrine) synthesis [62]. Prolonged repetitive physical exercises may activate signalling testosterone and brain-derived neurotrophic factor (BDNF)-dependent pathways, leading to a raise of tyrosine hydroxylase activity and increasing catecholamine levels [63].

ORN seems to have an antifatigue effect increasing the efficiency of energy consumption and promoting the excretion of ammonia [29, 64]. Some authors found that ORN promotes fatty acid and protein catabolism improving physical performance and fatigue tolerance, especially in female athletes [29].

Also, we observed a reduction in CIT, used with aspartic acid to synthesize arginine-succinate that is a precursor for arginine, the primary substrate for NO biosynthesis. These data could be explained by the elevate increase in NO production in BH-divers [37]. Indeed, NO plays a key role in the adaptation of subjects exposed to high hydrostatic pressure [14] and recent measurements taken in SCUBA and BH-divers at − 40 m depth showed remarkable increases in the plasma concentrations of NO derivatives [37, 44]. Particularly, NO is the principal molecule involved in the regulation of vasoconstriction/vasodilatation mechanism, necessary to adapt the endothelium to the increased ambient pressure and the related regional modifications [14].

On the other hand, the increase in pO_2_ triggers the formation of ROS and RNS leading to oxidative stress, [40]. SCUBA and BH-divers can activate the endogenous antioxidant system to control vascular oxidative stress [43, 65]. This can explain the raise of serum CYST concentration: CYST is an important Cysteine source that is, with GLU and GLY, necessary for glutathione biosynthesis. GLY reduction may be due to the synthesis of glutathione: this is also confirmed by the increase in the glutathione peroxidase whose main biological role is to protect the organism from oxidative damage [43, 66, 67].

Finally, hypoxia occurs during the final part of BH-diving (ascent phase) [10]. SER is involved in the protection from hypoxia: mitochondrial serine catabolism protects from hypoxia maintaining mitochondrial redox balance and cell survival [45]. PRO decrease may be also related to the production of GLY, involved in the synthesis of antioxidants. The production of TAU precursors and TAU would underlie the tolerance to hypoxia: some authors registered a dose-dependent protective effect of TAU on the synaptic function of rat hippocampal slices exposed to a hypoxic insult [68, 69]. A similar protection mechanism may occur also in BH-diving despite the intermittent hypoxia. TAU decrease seems to be related to its antioxidant properties protecting tissues from highly toxic hypochlorite produced by inflammatory cells in the course of free radical processes [70] and other oxidative stress markers [71].

Our data seem to indicate a clear picture of the body adaption to hyperbaric exposure in BH-divers. From the interpretation of these results, it is clear that energetic metabolic request (for physical effort and for body adaptation to the extreme environmental) is in large part supported by AA used as substrate for fuel metabolism. The reduction in several AA involved in energy support at T1 seems to influenced by the characteristic of BH-diving, probably related to the “relax and comfort” training, diving experience and diving techniques adopted by expert BH-divers. Despite the absence of data related to serum AA changes in BH-diving, the major part of BH-Divers seems to perform their repetitive dives without an intensive muscle effort due to the correctness of the athletic gesture, the use of appropriate equipment and the adequate mental technique. Furthermore, it could be interesting to extend this test in other BH-diving specialty (static and dynamic apnoea) in which the use of muscle is absent (static apnoea) to use this model to understand better AA changes in BH-divers.

The short-term effect of serum AA profile changes found represents the most important data in our results and may indicate a muscle activity more intense than that usually BH-divers perceive/referred.

Data related to the NO production and antioxidant synthesis could explain the well-known BH-diving-related vascular adaptation and the response to oxidative stress during diving deep phase, as observed by SCUBA and BH-diving underwater blood draw studies [37, 44]. Finally, there are interesting data related to the hypoxia [72, 73] stimulus that indirectly may confirm that the muscle apparatus works under strong exposure conditions notwithstanding the very short/low intensity of exercise, due to the intermittent hypoxia caused by repetitive diving.

Obviously, the AA catabolism may be also explained in part by the increases in circulating AA besides cardiac and skeletal muscle work in the particular muscle activity conditions (increase in pressure, hypoxia in ascent phase, diving response) requiring several adaptation mechanisms including smooth muscle-mediated massive vascular response.

Even if it is well known that water immersion affects fluid balance, causing a redistribution of blood volume and an increase in urine production which results in fluid loss (dehydration). Our BH-divers did not show differences in blood volume, calculated by the Dill and Costill formula, between pre- and post-diving. This might be explained by the fact that all the BH-divers were expert instructors and/or high-level athletes that common drink adequate amount of water during the BH-diving training session.

Our results about changes in serum amino acid profile after repetitive breath-hold dives are an absolute novelty in this specific field, as far as we know, and could represent an interesting new approach in the study of BH-diving physiological adaptation and become very interesting to structure BH-diving much more specific protocols, than it was done in the first preliminary study, allowing to better select the numerous stimuli that a diver undergoes.

### Limitations

The two main limitations of this study are the reduced sample size, and the absence of data related to NO production and oxidative stress changes which would have allowed a more in-depth analysis of the results. Furthermore, it could be interesting in the future to compare our data to those obtained by volunteers performing static and dynamic apnoea to analyse the different risk factor separately.

## Conclusion

Investigating serum AA changes after a BH-diving training session, we found a statistically significant decrease in AA involved in the field of energy need to sustain and to adapt to the physical effort. Furthermore, we found a significant reduction in CIT, involved in NO production to adapt the endothelium to the hyperbaric condition, and in AA related to antioxidant response, to protect from free radical damage. Finally, we observed a decrease in AA related to hypoxia adaptation that indirectly may indicate that the muscle apparatus works under strong effort notwithstanding the short/low intensity exercise perceived/reported by BH-divers. With the exception of CYS, the most of them returned to the basal value some hours later. Further tests will be necessary to better understand the origin of these AA changes, focussing separately on the different BH-diving protocols and related risk factors (muscle work, increase in pressure, hypoxia, diving response, environmental conditions).

## Data Availability

The raw data supporting the conclusions of this article will be made available by the authors, without undue reservation.

## Abbreviations

AA: Amino acid
ALA: Alanine
BH-diving: Breath-hold diving
CoA: Coenzyme A
CYST: Cystine
FFA: Free fatty acids
GLU: Glutamate
GLY: Glycine
HIS: Hystidine
ILE: Isoleucine
LEU: Leucine
LYS: Lysine
MET: Methionine
NO: Nitric oxide
ORN: Ornithine
PHE: Phenylalanine
PRO: Proline
SER: Serine
TAU: Taurine
THR: Threonine
Tyr: Tyrosine
VAL: Valine
